# Developing a Messaging Graphic for Storage Times of Refrigerated Ready to Eat (RTE) Foods for a Consumer Food Safety Health Campaign

**DOI:** 10.3390/ejihpe10030062

**Published:** 2020-09-01

**Authors:** Delores Chambers, Edgar Chambers, Sandria Godwin, Alisa Doan, Sheryl Cates

**Affiliations:** 1 Center for Sensory Analysis and Consumer Behavior, Kansas State University, 1310 Research Park Dr., Manhattan, KS 66502, USA; delores@ksu.edu (D.C.); ardoan@gmail.com (A.D.); 2 College of Agriculture, Human and Natural Sciences, Tennessee State University, Nashville, TN 37209, USA; adgsgrandma@gmail.com; 3 RTI International, 3040 East Cornwallis Road, Research Triangle Park, NC 27709, USA; scc@rti.org

**Keywords:** food safety, ready to eat foods, conjoint analysis, social marketing, education: RTE, health

## Abstract

This study developed and evaluated risk communication messages for ready to eat (RTE) foods targeted towards consumer storage practices in a food safety health campaign. Concepts were determined from a fractional factorial design of five categories of attributes potentially present in health promotion: title, message, graphic, slogan, and icon. Consumers viewed a subset of concepts and scored how useful the concept was in remembering to throw away RTE foods that were stored too long. Regression analysis determined which combinations of message attributes were most likely to result in using the information to throw out foods, which could help prevent foodborne illness. Findings showed that for this type of information, a graphic is a critical element for the printed schematic. The slogan (i.e., a short statement similar to a jingle or tag-line in a commercial) may be important to consumers, but the icon was not important.

## 1. Introduction

Changes in population demographics, the wide variety of foods we eat, and the global nature of our food supply are some of the factors that influence the prevalence of foodborne illness [1]. The continuing evolution of lifestyles has definite implications for what consumers eat, where food is obtained, how food is prepared and where food is prepared—all important dimensions for food safety. Van Loo et al. [2] showed that consumers chose ready to eat (RTE) foods because they were convenient, tasted good, and were perceived as nutritious and safe. That meshes with information from other researchers [3,4] who found that although liking was always the most important consideration in food choice, convenience and health were always in the top five reasons for choice at each meal or snack occasion. Thus, consumers’ increased reliance on foods that are convenient, portable, and quick/easy to prepare has led to increased consumption of RTE foods. RTE foods are usually consumed without further preparation by the consumer (i.e., without cooking or treatment that could kill bacteria); thus, it is important that these products are handled and stored properly. In lower and middle income countries RTE foods often are sold by venders and may be exposed to higher temperatures and contaminants such as insects and rodents [5].

RTE food products such as sausages, deli and luncheon meats, salads, smoked seafood, and soft cheeses represent a potential vector for transmission of foodborne pathogens such as *Listeria monocytogenes* [6,7] and *Salmonella* [8,9]. Recent research has shown the propensity for a number of different types of RTE products to show potentially problematic microbial contamination [10,11,12,13,14]

## 2. Literature Review

### 2.1. Consumer Food Safety Practices

Although many consumers might agree that food safety is important, whether those same people consciously consider food safety as they store, prepare, and eat food each day is far less likely. An increasing number of consumer studies have shown that consumers are not handling and storing food properly. For example, various studies tracking shopping, storage, or preparation in-home have shown that many consumers risk of foodborne illness from improper handling of food [15,16,17,18,19]. Even when preparing food at laboratory kitchen sites, consumers have shown that they often do not follow recommended practices [20,21,22], which can result in cross contamination. Previous studies have shown that consumers have misconceptions regarding proper food handling and refrigerator storage techniques including RTE foods [23,24,25]. Actual measurements in home refrigerators in the US and UK have shown that consumer refrigerators are not necessarily set at proper temperatures [26,27]. Changing consumer behaviors is a slow process, and consumers need good reasons to change longstanding behavior. Strategies for improving behaviors and lowering consumers’ chances of getting foodborne illness are needed [17,28,29,30,31,32].

### 2.2. Food Safety Education Programs for Consumers

Food safety education is a priority of government and consumer organizations [33,34]. Educating consumers is an important step in helping to prevent foodborne illness but information must be in a format that is meaningful or useful [35]. Government regulators and other communicators play an important part in getting information to consumers. However, Wilson et al. [36] found that communicators need a good understanding of consumer communication and have a desire to be more proactive in those communications. Preventing foodborne illness through improved consumer practices in the home requires research-based educational delivery that is appropriately designed to engage and motivate consumers and convey messages that are science based and consumer focused.

Determining the best way to educate consumers on food safety can be challenging but data show that many methods can work, including both in person and on-line educational programs, if the educational materials are appropriately developed [37]. However, some racial and ethnic differences have been noted for food safety messaging, which suggests that some people prefer more informational messaging, while others may prefer more guilt- or fear-inducing information [38]. A recent study showed that results can vary depending on the demographics of the participants in a major food safety campaign [39]. Similarly, authors have found differences in the ways in which information is provided in nutrition education displays among older and younger consumers and some ethnic groups [40,41]. Other authors have shown that many consumers preferred a simpler “more detached” approach to understanding food safety risks associated with food radiation risks [42].

Applying a social marketing framework can assist in making a change that consumers can follow clearly. Social marketing has been shown to be effective when used appropriately for food safety [43], but social marketing must identify the drivers of change [44]. It is imperative that strategies to drive change be visual, simple and easy to understand and remember [45] and “graphical messaging” can meet that need [46,47]. One issue is that there is a finite amount of space on a graphical display and a finite amount of information that consumers can quickly read and grasp. Common graphic messaging includes such items as food pyramids (plates, pagodas, triangle, etc.) and tobacco use warning labels.

### 2.3. Determining Trade-Offs

Because it is impossible to provide all the information that is desired by everyone, trade-offs of information must take place. This is especially true for small graphical elements that may be used in promotional materials (e.g., refrigerator magnets), on websites, one page fact sheets, presentation slides, etc. Thus, it is necessary to determine the value that consumers place on different product attributes, e.g., health promotion graphics/words. Furthermore, it is critical to determine which of those “attributes” consumers might eliminate to obtain other attributes that are more beneficial to them [48], e.g., particular pieces of information they want and need most.

One technique that is used to assess possible consumer impact of educational materials is conjoint analysis (CA), a form of discrete choice modeling. This method has been used widely to evaluate new products and product attributes in the marketing field, but also for determining messaging for fruit and vegetable consumption in patients at risk for heart disease [49], consumer health education plans [50], ways to promote health benefits of products [51,52,53] and better understanding of preferences for health services [54,55]. Ultimately CA allows the best achievable combination of characteristics to promote a particular positive behavior, concept, schematic, or product [56]. It can be used to estimate how important a characteristic is to the consumer and provides a “utility” or importance that shows the unique value of each element, characteristic, or message [57].

In health marketing campaigns the objective is to engage the consumer, provide a motivation for change, explain the change needed and how to do it effectively, and often provides an icon or link to other supporting information. In traditional marketing this is known as “the hook, the story, and the offer”. The messages themselves are a combination of many factual and structural elements. The consumer of the message depends on the message developer to provide content that is organized and emphasizes the overall message in the best way possible [58]. For graphical messages there are many components that make up the schematic but of critical importance is the structure, which is key to consumer understanding [59]. If one simply considers any “figure” in a journal article, which is a graphical message, there is a title (often the figure number), a message (the actual “title” of the figure with the information on what the figure contains, the graphic element that contains the data, and possibly one or more footnotes or explanatory elements. Of course, the actual design depends on the type of message that is needed. For this research the type of message is categorized as a “persuasion” message [60] meant to persuade people to do something. Scanning other types of persuasive health messaging campaigns suggests that a title, a message, a graphic, a slogan, and an icon (and sometimes additional link information) often are present in a single graphical element. For example, the downloadable magnet for the “Is it done yet?” campaign for safe meat cooking temperatures [61] contains a title “Is it done yet?”, which also serves as a slogan; a message “you can’t tell by looking …”; a graphical chart; a second message, “USDA Recommended Safe …”; Icons (the USDA icon, and four icons associated with food safety); and other information including telephone numbers, a website, and the name of a USDA agency.

The objective of this study was to design a simple educational graphic tool to be used as a part of other educational materials that could be effective at motivating consumers to throw away RTE foods that are past their shelf life. CA was used to determine which combination of attributes would most likely lead consumers to follow proper storage recommendations for ready to eat foods.

## 3. Methods

### 3.1. Consumers

Two-hundred forty United States (US) consumers, ages 18 and over, from Manhattan, KS, (population approximately 50,000 people) and Nashville, TN, (population of over 1,500,000 people) were recruited by telephone from existing consumer testing databases to participate in this study. Participating consumers must regularly have purchased 40% or more of the household’s food at a grocery store or supermarket at least once per month and stored it in their home or apartment refrigerator. The demographics of the eligible participants was collected *post hoc* and was: women (62%) and men (38%) from a range of racial and ethnic backgrounds: 69% white, 18% black, and 14% other races (including Asian, Native American, mixed race, or other); approximately 10% were of Hispanic ethnicity. Of the participants, approximately 40% had a gross household annual income of less than 25,000 USD. Ages ranged from 18 to 65+ with numbers within various age ranges distributed approximately evenly. All were native English speakers and were paid for coming to a 30 min testing session. The research was approved by the Institutional Review Board for Human Subjects at Kansas State University under protocol #5930.

### 3.2. Conjoint Analysis (CA)

CA has been used widely [62,63] to evaluate new products and product attributes in the marketing and sensory fields [64,65,66]. CA uses statistical techniques to model the impact of each specific characteristic on the overall “product”, which may be an actual product, a service, behavior, a promotional idea, or as in this study, a graphical element with various parts. Moskowitz [67] reported that consumers have an easier time evaluating concrete situations (i.e., whole concepts), rather than evaluating specific attributes for importance. Using CA allows consumers to respond to entire products/concepts and then uses the “utility scores” or “part worth” values of the individual components to build various combinations of products/concepts to determine what is “optimal”. Results from CA show consumer “ideals” or preferred concepts/products based on the characteristics presented. Thus, CA uses whole concepts to quantify which characteristics consumers are most interested in (positive utility scores) or are willing to sacrifice (negative utility scores) in order to get other attributes.

### 3.3. Concept “Cards”

**Figure 1:**
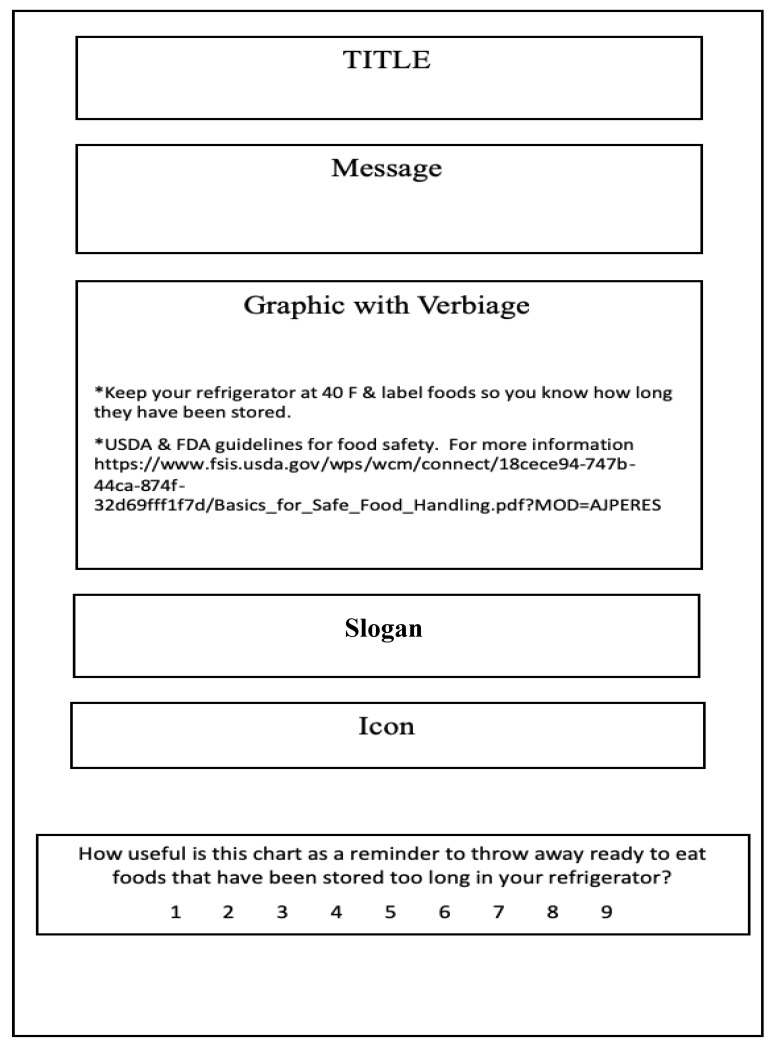
Outline of a Schematic shown to consumers (a title, message, graphic, slogan, or icon).

**Figure 2:**
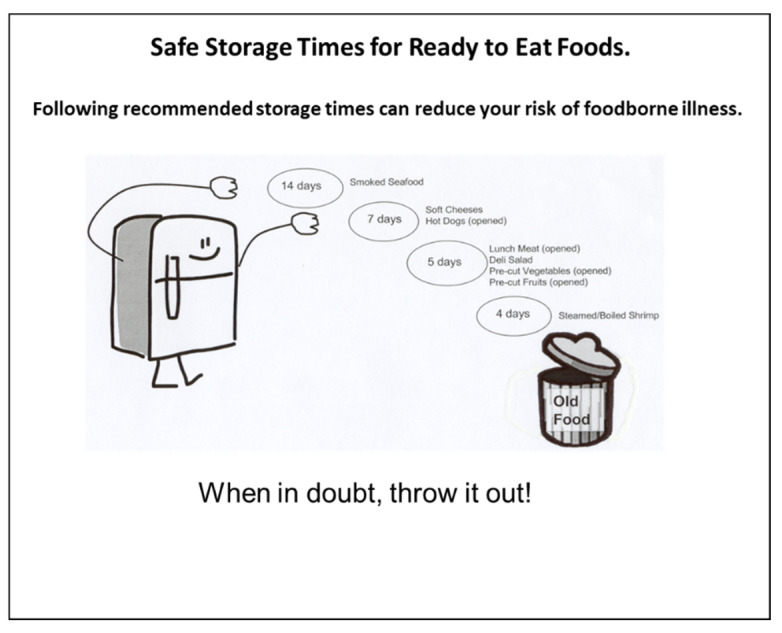
Example of an actual concept schematic (no icon is used in this example).

In CA, various whole product concepts are presented as a group of specific category attributes. For this study, concept graphics or “schematics” included attributes from each of five different categories (Figure 1 and Figure 2), i.e., title, message, graphic, slogan, and icon. Figure 1 is a diagram of how the concept schematic would be set up and Figure 2 shows an actual concept. Note, that the scale to evaluate the message also is shown in Figure 1. Recent research [68] using a similar CA approach (five categories, each with various attributes) to develop a label for a health-related attribute of products showed that a majority of consumers were influenced by some variables more than others. The categories in this project were determined through discussion with the researchers and two graphic artists with experience in developing social marketing campaign materials. Attributes were determined during in-person discussion by five researchers who had previous experience with health messages and prior publications related to consumer food safety issues. Initially, a large list of potential topics and statements were generated from literature reviews of previous messages on safety of ready to eat foods and leftovers, discussions with consumers, and personal expertise and discussions among researchers. Each topics, element, statement, or concept was put onto a card, the cards were discussed and grouped, and then the groupings were discussed to determine overlaps or whether any items were missing. After discussion, key message elements were determined and placed into a messaging category. Then each element was discussed and the list of elements within each category was narrowed to those used for this study. Discussions with consumers and extension food safety experts confirmed the face validity of the items selected. It must be noted that for some categories a “blank” or no attribute was tested to determine the necessity of that category.

### 3.4. Concept Categories and Attributes

The title of the schematic was the first category, and it was expected to identify the concept schematic’s main topic for the consumer. Because titles could be short or long, three titles with different approaches were taken to determine if a question-style title would be accepted more than an informative/descriptive title type (Table 1). One title was used in each schematic shown to consumers.

The next category was the message, which was used to relay the importance of knowing about RTE foods and their storage times to the consumers. The messages needed to be longer than the title but short enough to get the information across to the consumers quickly. A message on each concept schematic was shown to the consumers (Table 1), except on the concepts where no message was to be presented; which assumed that the graphic carried the message sufficiently.

**Figure 3:**
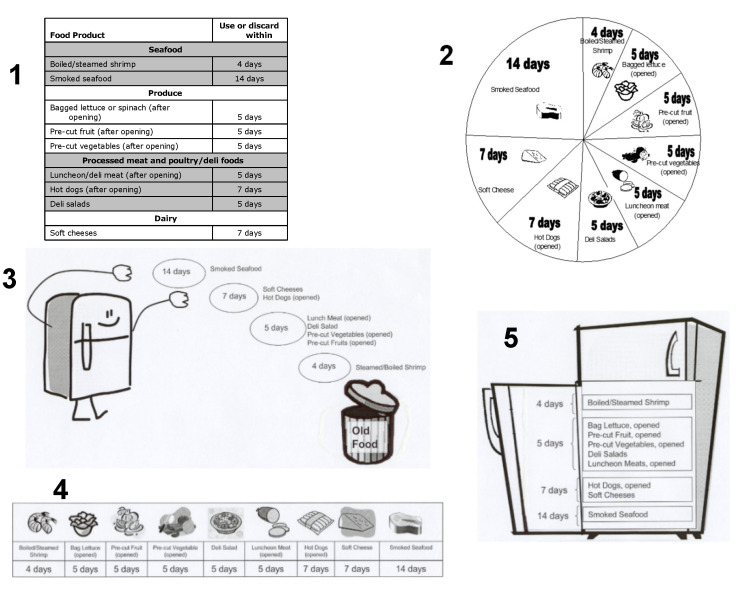
The Five Graphics Used in Graphics Category.

The third category was the graphic; each concept schematic had one graphic, which consumed the majority of space on each schematic because it contained the specific information the consumer needed to know in order to throw out food in a timely manner. The graphic was the part of the schematic that gave the most detailed information about how long RTE foods could be stored. Figure 3 provides the information that was contained in each of the five graphics and shows how each of the graphics looked Each graphic was centered in the concept schematic. The graphics were designed by a graphic designer to represent different possible approaches to the design: (1) tabular with typed foods, (2) wheel with visual foods, (3) action with typed foods, (4) bar with visual foods and (5) visual representation of the refrigerator with typed foods. In all the graphics the days were typed (numerals) and were listed in a day order in each graphic.

The fourth category was the “slogan” (Table 1). Slogans are usually short, often use sound or are easy to say or remember, and are used to catch the attention of the consumer with something they can remember [69,70]. In this context it was viewed as a quick thing for consumers to remember and say to themselves as they look through their foods. Because little research has been conducted on jingles (or slogans), Taylor commented that “more research on this topic would be a welcome development”. The option of “no slogan” was included to determine if a slogan was necessary for the schematic.

**Figure 4:**
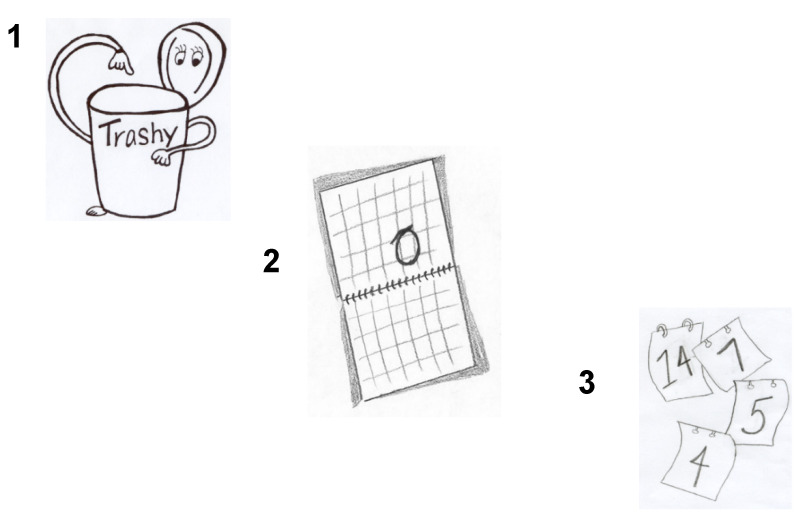
The Three Icons Used in Icon Category.

The last category was the icon. Icons typically are used with other messages, e.g., hand-washing and proper cooking temperatures for meat [71]. Using an icon in marketing (in this case, social marketing of food safety habits) can help people immediately associate a message with a certain product or habit. Icons also can help speakers of different languages understand what is being asked or explained without having to know what the exact words are on the schematic. For example, the hand washing icon mentioned earlier clearly indicates that hand washing is expected. Figure 4 shows icons that were present on some concept schematics. As with the message and slogan, not all schematics showed an icon in order to determine if an icon was needed.

### 3.5. Consumer Test Procedure

Consumers viewed printed schematics on cards and answered the question “How useful is this chart as a reminder to throw away ready to eat foods that have been stored too long in your refrigerator?” by marking the appropriate number on a numerical scale ranging from “1 = not very useful” to “9 = extremely useful”.

A fractional factorial design of 240 schematics was taken from a 3 × 6 × 5 × 6 × 4 factorial (the number of title options X the number of message options X the number of graphic options X the number of slogan options X the number of icon options) to provide the ability to compare main effects of category and 2-way interactions of categories as well as study the individual elements within categories. The 240 concept schematic cards were divided randomly into 10 groups of 24 cards each.

A group of 24 concepts was randomly assigned to a particular consumer, and each consumer evaluated the 24 concept schematics in random order. Gofman and Moskowitz (2010) discuss the use of such experimental designs in CA. Consumers could take as long as they wanted to look at and read each card and then mark the scale. After marking the scale they went to the next concept. Pretesting suggested that consumers would take 15–20 min to complete the task. Thus, consumers were told they were needed for 30 min to ensure each person had plenty of time to finish. No one took longer than that to complete the task. Because the task was relatively simple (read and look at a group of concepts with some repeating attributes and respond to one question per concept), no concerns with fatigue were expected.

### 3.6. Data Analysis

Scores for each of the 240 schematics were used in a regression procedure using maximum likelihood modeling (SAS 9.1, SAS Institute, Cary, NC, USA) to calculate the “part worth utility values” for each attribute. The “part worth utility value” is the regression coefficient for that particular attribute or the “level” that the attribute increases. or decreases the overall score [72]. In the regression modeling based on a dummy variables process, each individual “attribute” is included as a component in the model; the overall category is not part of the model. The values of the model take either a 0 or 1 when an attribute is present and only one attribute within a category can be present at any given time. This procedure is used to predict the additive value that any particular attribute (e.g., “Storing foods too long can make you or your children sick” or the icon of the trash can called “trashy”) gives in determining if that attribute is a useful reminder to consumers to discard RTE foods that likely are past their shelf-life. The more positive the utility score, the more that attribute is viewed by consumers as a helpful reminder to them for throwing out food. Larger, negative scores would imply the attribute is providing “clutter” in the message or detracts in another way from the overall message and should not be used. Within a category, Tukey’s was used to determine significant differences (*p* ≤ 0.05) among utility scores. After determining the regression equation using only the individual components, 2-way interactions of components also were included to determine the impact of interactions on the part worth scores. There were no 2-way interactions. Higher-level interactions were not included because impact of the incomplete block structure precluded such analysis.

In addition to the part worth utility score, conjoint analysis allows the computation of the relative importance of each category of attributes. The relative importance is based on the range of part-worth utility scores for a category divided by the sum of the range of those values and is calculated as a percentage [48,73].

## 4. Results and Discussion

### 4.1. Relative Importanc of Categories for the Graphical Schematic of Message

The importance of each category of attributes is shown in Table 2. Graphic was overwhelmingly the most important category with slightly more than half of the total percentage of importance. Slogan and Message were found to be the next most important categories using the Relative Importance Scores (a percentage). Title and Icon appear to be the least important aspects of the graphical schematic. It must be emphasized that the method of determining importance only considers the difference in impact of the individual components within the category and not the essential nature of the category. For example, would it be clear what the graphic was about if it did not have a title? This was considered for three of the categories (message, slogan, and icon) but not for the itle or gaphic categories. That likely would impact the relative importance of the title because the relative importance of Message and Icon would apprear to be far less if the “no message” or no icon” attribute were eliminated.

These findings show that the graphic is essential to the Graphical Schematic in this study. Other aspects such as the message and the slogan appear to be potentially important but may depend on which message or slogan is used. Title and Icon are of lesser importance in this study.

### 4.2. Individual Attributes of the Graphical Schematic

Table 3 shows the utility scores (i.e., standardized regression coefficient) for each attribute within a category and whether significant differences were noted between those attributes for the category. Graphic was the only significant (*p* ≤ 0.05) main category effect from all five categories (title, message, graphic, slogan, icon) of schematic attributes, although we chose to show that slogan also provided a trend toward significance. This overall effect indicates that a specific graphics could produce a positive effect on intended behavior. Providing a graphic is critical both to providing the information needed by the consumer in a simple format [42] and also provides a meaningful “anchor” that is needed by consumers to help mitigate perceptual bias that can underestimate food safety risk [74]. Because the graphic was the most prominent feature of the schematics viewed by consumers, its importance, relative to other components in the overall message, could be explained easily. Graphic 4, the bar style, increased the overall score most while Graphic 3 and Graphic 5 decreased the overall score the most. Open-ended comments from consumers suggested that the two heavily graphic designs (3 and 5) may have been too cluttered and the message got lost, but no formal analysis was conducted on that qualitative data. Perhaps because Graphic 3 and Graphic 5 were more complex than the other graphics, they may have required more time to figure out what the information was trying to relate. In contrast, the bar graphic (Graphic 4) had positive comments related to how simple and easy it was to understand and that it seemed like a familiar format. A few comments mentioned that it looked somewhat like a calendar of days, which might “make sense”. However, the calendar-like comments could show that the format could potentially be misleading because some of the boxes have the same number of days and consumers could get confused thinking that foods further down the bar automatically have longer storage times. This would need to be tested in further research.

Although no significant effects were found within any other main effects, some attributes did provide slightly positive utility scores while others were clearly more negative. Results depended on the specific title, message or slogan. For example, Title 2, “Safe Storage Times For Ready To Eat Foods,” resulted in a marginally higher utility score (i.e., increased likelihood that its use would contribute to consumer use of the concept for throwing out old RTE foods) than title 1, “Recommended Storage Times For Ready To Eat Foods,” or title 3, “How Long Can I Keep It,” which both had negative individual utility scores. Comments suggested that all of them were rather uninteresting titles, but that Title 1 was particularly dull and that Title 3 was rather vague and did not specify food. These are important aspects to remember for future research. Consumers need to be engaged by health promotion messaging [75] and boring titles will not accomplish that goal. Titles that have little to offer in terms of being appealing probably contribute to the low score for relative importance.

Message 1, “Storing foods too long can make you or your children sick,” message 2, “Short storage times will keep refrigerated foods safe to eat,” message 3, “FDA & USDA have recommended storage times for ready to eat foods,” and message 4, “Following recommended storage times can reduce your risk of foodborne illness,” were more positive than message 5, “Do not store foods longer than recommended,” and message 6, which was no message. As with some of the other main categories, the differences among attributes were not significant, but do show some informational trends. Of note is that the lack of a message provided a negative utility score and the score was more than three times more negative than the statement with a negative utility score in that category. The information from this category becomes more apparent when the open-ended comments are considered. Messages that easily speak to safety, have a reputable source as background, or for some people invoke fear may have more of an impact than graphical schematics that simply state ”do” or “do not” do something without a reason (message 5) or that have no message at all (Message 6—none—in this test).

For the slogan category, slogan 1, “When in doubt, throw it out,” and slogan 2, “How long is it safe,” were found to be more likely to result in positive behavior change (based on consumer’s responses) than slogan 3, “Use it or throw it,” slogan 4, “Eat it or toss it,” slogan 5, “Toss it or toss it,” and slogan 6, which was no slogan. It should be noted that these typically were trends (*p* < 0.10) that did not meet the more stringent significance of *p* ≤ 0.05 for these categories. However, comments indicated that “When in doubt, throw it out” has been used for a number of years already and was familiar to some people. In addition, that slogan also uses several traits associated with memorable and successful slogans including techniques such as assonance and alliteration, ellipsis, parallelism, and rhyme [76]. “How long is it safe” was a question that made some people “take a second look”. The others received comments ranging from “wasn’t memorable at all” to “didn’t really understand it” to “just gross when I figured it out”. The use of easy to use aids that jog memory has been shown to help people remember some details better [77,78,79,80].

The icon category produced no differences, either significant or trends. This suggests one of three things: (1) icons had little impact on the consumers’ feelings about the overall message, (2) consumers did not particularly care for any of the icons, or (3) needing an icon for recognition is acquired over a period of time and is not conducive to short-term study.

There were no significant 2-way interactions found in the data. Interaction effects (e.g., the impact of a particular category on another category) can increase or decrease the score for the schematic. In this case, an interaction of the various categories was not necessary for developing a good schematic that consumers believed could help them change behavior. This is an important finding for this study because interactions can make results more complex to interpret and sometimes more complex to implement. In this case, the results were clearly dependent solely on the individual attributes of the concepts and not on the dual interactions of concept elements such as the interaction of slogan and message or title and graphic.

### 4.3. Demographic Subgroup Information

The wide range of consumer demographics including gender, age, race, location, and income made it possible to identify several subgroups. In general, the results of all of the possible subgroups were similar to the overall results with a few exception. The younger age group 18–24 (n = 45), minority consumers from the large metropolitan area (n = 39), and low-income consumers from the large metropolitan area (n = 34) preferred graphic 1, the table format over graphic 4. This could be because of consumer familiarity with that tabular graphic format as a few respondents mentioned. However, the subgroup samples are so small that it also could simply be an artifact of the small samples sizes and over analysis of those small samples.

### 4.4. Overall Graphical Schematic—Maximum Score

**Figure 5:**
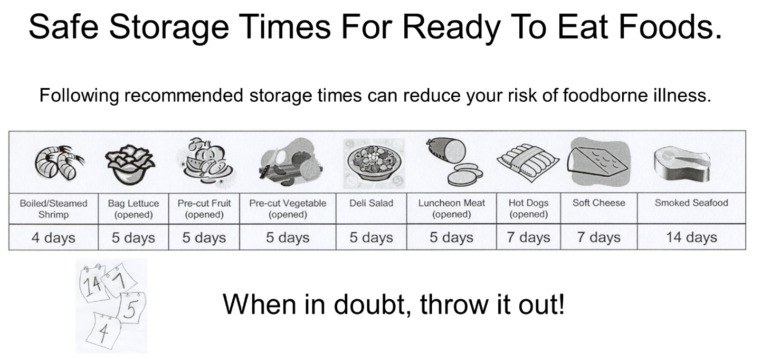
Highest scoring (+1.0) graphic for use with consumer messaging on safe storage times of Ready to Eat (RTE) Foods.

Because no interactions were found calculating the standardized score for any combination of elements or attribute is quite easy. The utility value for each attribute found in a graphical schematic can be added together to determine the “score” for that schematic. Thus, in this case, the highest potential score (+1.0) is shown in Figure 5 and includes Title 2, Message 4, Graphic 4, Slogan 1 and Icon 3. The lowest potential score (−0.98) would be a combination of Title 1, no message, Graphic 3, slogan 5 and no icon (Recommended Storage Times For Ready To Eat Foods; the refrigerator throwing food in the trashcan, Toss it or Toss it!).

Because the graphic was by far the most important category and the only statistically significant (*p* < 0.05) category in terms of a regression coefficients, use of a graphic is necessary to communicate important information with other categories of information in the message present only to support what is displayed. Title would be a needed element only to identify the Graphical Schematic if the message cannot stand in its place. Message appears to be a needed element (Relative Importance 17%), but additional research is necessary to determine if combining the message and the title into one short phrase may represent both. In the final Graphical Schematic,, the “title” stating “Safe Storage …” may be able to be eliminated (with the message being “promoted” to title status) to provide an even more streamlined graphical element in social media marketing for safe food storage of RTE foods. The slogan also appears to be important (Relative Importance 19%) and also may be important because it provides a quick short “memory cue” that may help consumers to be more conscious of safe storage for food. Consumers in the study did found the icon category to be relatively unimportant but further research is needed to determine if that is because of the icons they tested, other issues, or whether icons simply are not important in the overall graphical message.

**Figure 6:**
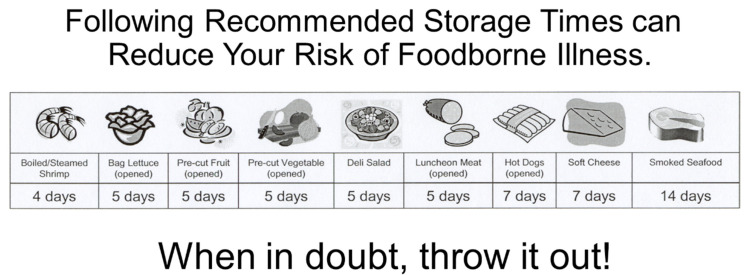
Simplified high scoring graphic (+0.87) for use with consumer messaging on safe storage times of Ready to Eat (RTE) Foods.

A simplified schematic is shown in Figure 6 and may score at or near +0.87 (Note that the absence of a title was not tested and, thus, we do not know the actual effect on the score). That schematic uses the message as the title, the main graphic and the slogan. This simplified graphic may be a better choice even though it theoretically scores lower because it may be quicker and easier read and understand and could take less space on items such as magnets, handouts, or websites to promote safer food storage. This is important because other authors [28] have suggested that providing consumers with or aids that simplify and enhance behavioral control, reduce barriers, and describe risk may be more effective than simply providing knowledge.

## 5. Limitations and Future Research Needs

Several aspects of this research limit its findings and potential interpretation. First, a reasonably small group of consumers was tested. Although each specific concept attribute (e.g., any particular title, graphic, etc.) was seen by every consumer, and each pairwise combination of concept elements was seen by more than 100 consumers, each of the 240 complete concepts was only seen by 24 respondents. Multiple authors have suggested a larger number of consumers be used in consumer sensory and marketing research studies [81,82,83,84]. Although there were no interactions noted, that low number of whole concept views can still limit the overall findings particularly when it comes to demographic subsets that would, of course, result in even smaller numbers. It is worth noting, however, that only small differences in demographic subsets were noted, which suggests that in this case, an overarching campaign graphical schematic may work generally for the total population.

In addition, this research studied the *content*, but not other aspects of schematics that can be further developed (e.g., the addition of appropriate colors and font styles) and distributed to consumers to potentially help improve the storage practices of RTE foods.

Further in-home testing is needed to determine if the graphical schematic developed in this research actually changes behavior related to using up RTE foods before their safety expiration date or throwing it out after such dates. This research measured consumers′ perception of their intent to change behavior, a commonly used surrogate for social marketing campaigns in areas of long-term change, but one that certainly does not actually indicate behavior change.

## 6. Conclusions

The study shows that a graphic that includes the essential information was by far the most important part of the graphical educational tool to be included in this health messaging campaign. In addition, both the message and slogan were noted as somewhat important by consumers probably to enable them to identity the education topic and provide a memorable aspect to enhance use. Titles and icons that might provide an overall identity to a campaign generally were considered nonessential by consumers for the graphical schematic educational tool in this research. Providing educational tools that can be used easily by consumers is important. In this case such tools used in campaigns have to potential to help consumers remember to discard food in a timely manner and help prevent foodborne illnesses caused by storing RTE foods longer than recommended. The study provides a sample process that could be used to develop focused schematics for health campaigns or other aspects of social marketing for health.

## Figures and Tables

**Table 1:** Titles, Messages, and Slogans Used for Schematics (See Figure 1).

**Titles**	
Title 1	Recommended Storage Times For Ready To Eat Foods
Title 2	Safe Storage Times For Ready To Eat Foods
Title 3	How Long Can I Keep It?
**Messages**	
Message 1	Storing foods too long can make you or your children sick.
Message 2	Short storage times will keep refrigerated foods safe to eat.
Message 3	FDA & USDA have recommended storage times for ready to eat foods.
Message 4	Following recommended storage times can reduce your risk of foodborne illness.
Message 5	Do not store foods longer than recommended.
Message 6 *	None
**Slogans**	
Slogan 1	When in doubt, throw it out!
Slogan 2	How long is it safe?
Slogan 3	Use it or throw it?
Slogan 4	Eat it or toss it!
Slogan 5	Toss it or toss it!
Slogan 6 *	None

* None means that no verbiage from that category was included in the “whole” concept schematic.

**Table 2:** Relative Importance for each Category within the Graphical Schematic.

Category	Relative Importance, %
Title	3.8
Message	17.1
Graphic	51.2
Slogan	18.9
Icon	9.1

**Table 3:** Utility Scores and significance of those scores in the Conjoint Analysis ^1^.

Category	Attribute Label from Schematic	Utility Score for Category Attribute	Attribute Differences
Title	Title 1	−0.0260	NS
	Title 2	0.0496	
	Title 3	−0.0236	
Message	Message 1	0.0138	NS
	Message 2	0.0626	
	Message 3	0.0900	
	Message 4	0.1173	
	Message 5	−0.0640	
	Message 6	−0.2197	
Graphic	Graphic 1	0.0873b	<0.0001
	Graphic 2	−0.0158c	
	Graphic 3	−0.4613c	
	Graphic 4	0.5501a	
	Graphic 5	−0.1602c	
Slogan	Slogan 1	0.2031	<0.10
	Slogan 2	0.0833	
	Slogan 3	−0.034	
	Slogan 4	−0.0724	
	Slogan 5	−0.1697	
	Slogan 6	−0.0104	
Icon	Icon 1	−0.0191	NS
	Icon 2	0.0397	
	Icon 3	0.0796	
	Icon 4	−0.1001	

^1^ Significance does not refer to whether the category is significant or not significant; it only refers to the differences among the attributes within the category. NS = Not Significant. a,b,c Graphics with different letters are significantly different from each other.
